# Body length rather than routine metabolic rate and body condition correlates with activity and risk‐taking in juvenile zebrafish *Danio rerio*

**DOI:** 10.1111/jfb.13100

**Published:** 2016-09-12

**Authors:** G. Polverino, D. Bierbach, S. S. Killen, S. Uusi‐Heikkilä, R. Arlinghaus

**Affiliations:** ^1^ Department of Biology and Ecology of Fishes Leibniz‐Institute of Freshwater Ecology and Inland Fisheries (IGB) Muggelseedamm 310 12587 Berlin Germany; ^2^ College of Medical, Veterinary and Life Sciences, Institute of Biodiversity, Animal Health and Comparative Medicine University of Glasgow Glasgow G12 8QQ U.K.; ^3^ Division of Genetics and Physiology, Department of Biology University of Turku Itäinen Pitkäkatu 4 Turku 20014 Finland; ^4^ Division of Integrative Fisheries Management, Faculty of Life Sciences Humboldt‐Universität zu Berlin Unter den Linden 6 10099 Berlin Germany

**Keywords:** allometry, behavioural type, body size, boldness, metabolism, personality

## Abstract

In this study, the following hypotheses were explored using zebrafish *Danio rerio*: (1) individuals from the same cohort differ consistently in activity and risk‐taking and (2) variation in activity and risk‐taking is linked to individual differences in metabolic rate, body length and body condition. To examine these hypotheses, juvenile *D. rerio* were tested for routine metabolic rate and subsequently exposed to an open field test. Strong evidence was found for consistent among‐individual differences in activity and risk‐taking, which were overall negatively correlated with body length, *i.e.* larger *D. rerio* were found to be less active in a potentially dangerous open field and a similar trend was found with respect to a more direct measure of their risk‐taking tendency. In contrast, routine metabolic rate and body condition were uncorrelated with both activity and risk‐taking of juvenile *D. rerio*. These findings suggest that body length is associated with risk‐related behaviours in juvenile *D. rerio* for which larger, rather than smaller, individuals may have a higher risk of predation, while the role for routine metabolic rate is relatively limited or non‐existent, at least under the conditions of the present study.

## Introduction

Individual animals within a given species commonly exhibit consistent differences in behavioural traits such as activity, risk‐taking, exploration, aggressiveness and sociability, referred to as animal personalities or behavioural types (Réale *et al.*, 2007). Theoretical and empirical studies suggest that animal personality is ultimately caused by ecological and evolutionary adaptation to ecological gradients and corresponding selection pressures, such as risk of predation (Wolf *et al.*, 2007), resource availability (Biro & Stamps, 2008) or social environment (Pruitt & Riechert, 2011). Individuals do not only differ in their experienced extrinsic conditions but also in intrinsic factors such as body size and metabolic (*i.e*. energetic) demands, both of which can also promote consistent differences in behaviour among animals and are commonly referred to as ‘state variables’ (Biro & Stamps, 2010; Wolf & Weissing, 2010; Sih *et al.*, 2015). A state variable represents any measurable trait that varies dynamically over time, often influencing the costs and benefits associated with a particular behaviour [*e.g*. age, size, body condition or physiological condition (Wolf & Weissing, 2010)]. The optimal behavioural response of an individual then depends on the interplay between both (often stochastically varying) extrinsic and intrinsic conditions in light of trade‐offs (Carere & Maestripieri, 2013), which in turn may lead to consistent among‐individual differences in expressed behavioural phenotypes and maintain behavioural variation in natural populations (Réale *et al.*, 2007; Killen *et al.*, 2013; Sih *et al.*, 2015).

Behavioural plasticity allows animals to rapidly adapt to changing ecological and state conditions on very short time scales to safeguard access to resources and mates and avoid predation (Ahrens *et al.*, 2012; Buskirk, 2012). For example, as physiological and morphological states change (*e.g*. hunger levels, body condition or size) it is likely that the risk‐taking propensity of individuals also changes (Krause *et al.*, 1998; Brown & Braithwaite, 2004; Brown *et al.*, 2007). In this context, hungry fishes, fishes with small body size and low energy reserves should be more inclined to explore potentially dangerous areas and take risks in the attempt to search for food than well‐fed, large individuals and those with a good nutritional state (Krause *et al.*, 1998; Brown & Braithwaite, 2004; Killen *et al.*, 2011). Energy reserves are generally known to affect animal behaviour (Uchmanski, 1985; Filby *et al.*, 2010), resulting in consistent foraging, social and risk‐taking responses of individuals characterized by varying energetic states (Clark, 1994; Brown *et al.*, 2007; Dial *et al.*, 2008; Filby *et al.*, 2010). In contrast, the effects of body size on activity and risk‐taking behaviour have not been consistent in the literature. For example, large individuals of some fish species have been reported to be more active (Kobler *et al.*, 2008) and bolder (Grant, 1990; Polverino *et al.*, 2016) than smaller individuals, and these larger individuals thus tend to more readily expose themselves to potential predators (Johnsson, 1993), probably because the risk of predation typically declines with increasing body length and height (Lorenzen, 2000; Nilsson & Brönmark, 2000). In other fish species, smaller size classes were reported to be generally bolder (Krause *et al.*, 1998; Brown & Braithwaite, 2004) and to show higher activity in the wild (Landsman *et al.*, 2015). One explanation for these inconsistent findings may rest in the size‐dependency of predation risk. In small‐bodied species it is possible that larger, rather than smaller, individuals may be under greater risk of predation because these larger individuals are preferentially consumed due to the larger absolute amount of energy and nutrition they offer for predators compared to smaller conspecifics (Persson *et al.*, 2003; Johansson *et al.*, 2004). From an ecological and evolutionary perspective, it would then be beneficial to decrease exploration rates in unsafe environments and risk‐taking as body size increases, as found for various poeciliids (Laland & Reader, 1999; Brown & Braithwaite, 2004).

In addition to state‐effects on behaviour, animal personality has also been related to consistent individual variation in maintenance metabolism, with spill‐over effects on the expression of life‐history traits, commonly expressed as the pace‐of‐life‐syndrome hypothesis (Réale *et al.*, 2007, 2010; Wolf & Weissing, 2010; Dwyer *et al.*, 2014). In fishes, higher metabolic demands are expected to promote foraging (Krause *et al.*, 1998), which in turn usually involve exposing oneself to predators and overall risky situations (Ahrens *et al.*, 2012), providing a link between routine metabolic rate (RMR), activity and risk‐taking (Metcalfe *et al.*, 2016). A commonly expressed hypothesis is that individuals with a high RMR should also be consistently bold (Biro & Stamps, 2010; Killen *et al.*, 2013), manifesting in a high propensity to take risks as a function of the elevated energy demand to satisfy. An alternative perspective is that bold fishes may also be highly active and therefore develop a high RMR to be able to sustain the high muscular activity and the cellular machinery (*e.g*. mitochondria) needed to maintain a high scope for activity (White & Kearney, 2013). Independent of the exact mechanism, any correlation between metabolic rate and boldness is expected to in turn affect individual life‐history productivity by carrying over effects on growth, maturation and reproduction (Biro & Stamps, 2010; Guenther & Trillmich, 2015).

Several recent papers, however, have questioned the generality of the correlation between routine metabolism and behaviour (Killen *et al.*, 2013; Mathot & Dingemanse, 2015). In fact, it has been suggested that the covariance between RMR and activity or risk‐taking is only revealed in environments that physiologically and ecologically challenge individuals to express ecologically relevant behaviours (Killen *et al.*, 2012). A key consideration is that many physiological rates, including metabolic rates, scale disproportionately with size (Uchmanski, 1985). Hence, variance in body size can significantly affect a cascade of physiological (Clarke & Johnston, 1999; White & Kearney, 2014) and behavioural traits (Clark, 1994; Dial *et al.*, 2008; Filby *et al.*, 2010; Healy *et al.*, 2013). As a result, differences in body size can strongly affect phenotypic differences among individuals and potentially override differences in behaviour stemming from variation in basic metabolic demand. Generally, studying cause–effect relationships among behaviour, metabolic rate and body size remain controversial (Oikawa *et al.*, 1991; Metcalfe & Monaghan, 2001; Nussey *et al.*, 2007), which motivated the present study.

This study contributes to the literature on metabolism and behaviour (Metcalfe *et al.*, 2016) by reporting correlations between routine metabolism and activity and risk‐taking in juvenile zebrafish *Danio rerio* (Hamilton 1822), while controlling for body size and body condition in individuals of the same cohort. The rate of routine oxygen consumption (Schurmann & Steffensen, 1997) and boldness‐related behaviours (Réale *et al.*, 2007) were characterized using intermittent‐flow respirometry and open field tests, respectively. Based on the body of literature reviewed above, the following predictions were explored: (1) individuals differ consistently in activity and risk‐taking behaviours and (2) these differences are explained by differences in state variables like body condition, body length and routine metabolism.

## Materials and methods

### Study organism and maintenance

Experimental animals (*n* = 68) were laboratory‐raised *D. rerio* [mean ± s.d.; total length (*L*_T_) and mass = 22·4 ± 2·9 mm and 0·105 ± 0·04 g] of the same cohort. All *D. rerio* were F10 generation descendants of wild‐caught individuals originally sampled in a river system west of Coochibar [West Bengal, India (Whiteley *et al.*, 2011)]. Before experimentation, *D. rerio* were housed in opaque tanks (320 l each) connected to a single recirculation system at 27° C with 14L:10D, which reflected the circadian rhythm of the species (Cahill, 2002). *Danio rerio* density never exceeded 1·1 individual l^−1^. *Danio rerio* were fed five times per day with a combination of *Artemia* sp. nauplii and commercial flake food (TetraMin; http://www.tetra-fish.com; 47% protein, 10% fat). Water quality was monitored regularly for temperature (mean ± s.d.; 27·01 ± 1·23° C), pH (mean ± s.d.; 8·41 ± 0·11) and daily for oxygen content (mean ± s.d.; 8·00 ± 0·21 mg l^−1^). A week before the experiment began, *D. rerio* were transferred to 30 l tanks located in a temperature‐controlled room. Here, external disturbances were minimized, while water temperature was maintained at a constant 25° C. *Danio rerio* density and feeding were maintained as described before.

### Metabolic rate assays

*Danio rerio* were fasted for 24 h prior to being transferred individually into 5 ml glass respirometers [Loligo Systems; http://www.loligosystems.com (White & Kearney, 2014)]. The RMR was then estimated as the minimum rate of oxygen consumption. Pilot trials, in which *D. rerio* were left overnight in respirometers for 18 h, determined that the metabolic rate of juvenile *D. rerio* stabilised after 2 h in the chambers. Thus, the time that *D. rerio* spent individually in the respirometers was minimised because extended isolation periods are known to negatively affect both health of social animals and hence data reliability (Seeman & McEwen, 1996). During the experimental trials, *D. rerio* were given a 2 h acclimation period, after which RMR, *i.e*. the minimum cost of maintenance measured at a particular temperature and post‐absorptive state while also allowing spontaneous activity, of each individual was taken as the mean level of metabolism over the subsequent 3 h period (Steffensen, 1989). Notably, the metabolic measures performed here did not allow disentangling the extra oxygen consumption beyond standard metabolic rate caused by spontaneous motion activity. Therefore, such measures are defined as RMR to account for the presence of intrinsic factors that may have altered the individual cost of self‐maintenance and activity (Schurmann & Steffensen, 1997).

An array of eight cylindrical glass chambers was utilized to perform measurements on eight *D. rerio* simultaneously. The chambers were submerged in an aerated water bath maintained at a constant temperature of 25·0° C, range ±0·1° C using a thermostat. Water oxygen content within the chambers was measured once every 2 s using optodes (Firesting 4‐Channel oxygen meters, Pyroscience; http://www.pyro-science.com). After placing the chambers into the water bath, the system was covered with opaque polystyrene to minimize disturbance during measurements. The entire apparatus was located within a second temperature‐controlled room comparable with the one mentioned above for the housing of the holding tanks. This experimental controlled room remained closed during measurements.

The oxygen‐saturated water from the water bath was periodically flushed into each chamber of the respirometer through an external pump that was set to turn on and off for alternating 15 min periods. The decrease in oxygen content in the closed chambers was measured at intervals of 2 s for each 15 min period (*i.e*. closed phase). Subsequently, each chamber was automatically flushed with aerated water for 15 min before the start of the next measurement. For each closed phase, specific oxygen consumption of *D. rerio* was calculated by using linear least squares regression (decrease in oxygen concentration over time expressed as mass‐specific RMR in the unit mg O_2_ kg^−1^ h^−1^). The first and final 2 min were excluded from each measure such that only the linear component of O_2_ degradation was captured (Dupont‐Prinet *et al.*, 2010). For each individual, six slopes of oxygen decrease were recorded, and the mass‐specific RMR was calculated as the mean of the final three measurements. During each experimental session, one of the chambers measured the oxygen decline in blank conditions without a *D. rerio* in the chamber. As a result, a simultaneous and consistent measurement of microbial respiration was obtained and subtracted from the estimation of *D. rerio* oxygen consumption provided by the system. The oxygen consumption measured in this study was similar to standard metabolic rates observed previously in 2 month old juvenile *D. rerio* of similar *L*_T_ (Lucas *et al.*, 2014). Because (mass‐specific) RMR was correlated with *L*_T_ (Table I), the residuals of RMR from a regression of log_10_‐transformed (mass‐specific) RMR *v*. log_10_‐transformed *L*_T_ were used to derive a size‐controlled index of RMR.

**Table I:** Pearson correlation coefficients of fixed factors in juvenile *Danio rerio*. Values above the diagonal represent the correlation coefficients between pairs of fixed factors, while values below the diagonal represent their *P*‐values. Note that residuals from the log_10_‐transformed mass‐specific routine metabolic rate (RMR, mg O_2_ kg^−1^ h^−1^) and log_10_‐transformed Fulton's *K* (g mm^−3^ × 100 000) were both independent of total length (*L*_T_; mm) and used for the statistical analysis

	*L*_T_	Fulton's *K*	RMR
*L*_T_		−0·142	−0·442
Fulton's *K*	>0·05		−0·117
RMR	**<**0·001	0·174	

### Behavioural assay to assess activity and risk‐taking

After the completion of the metabolic measurements, *D. rerio* were housed individually in 5 l transparent tanks (25 cm × 15 cm × 15 cm) for 24 h before conducting the behavioural experiments. Because of the importance of visual cues in *D. rerio* behavioural responses (Polverino *et al.*, 2012; Kopman *et al.*, 2013), visual interaction between individuals during the 24 h of isolation was allowed to minimize stress on the focal individuals induced by isolation. Activity and risk‐taking of each *D. rerio* were tested individually in a standard open field test (Ariyomo & Watt, 2012) during two distinct measuring events (referred to as the first and second trial) to assess the repeatability of these two behavioural traits. The open field test is widely utilized to characterize activity rates and the individual position in the shy‐bold continuum for teleosts and it is based on the assumption that a novel, open and structureless field is considered dangerous. Under this perspective, highly exploratory individuals or those that move especially in the central danger zone might be more willing to take risks (Burns, 2008; Ariyomo & Watt, 2012). The circular experimental arena (48·5 cm in diameter) used in the present study was covered with white contact paper on its bottom and lateral surfaces to guarantee a high colour contrast and to facilitate *D. rerio* identification. Notably, the colour of the contact paper used for the experimental arena matched the internal colouration of the housing tanks where *D. rerio* were housed since birth. In this way, stressful responses of individuals to the high contrast background of the experimental arena were prevented. Two neon tubes on the room ceiling assured standardized light conditions between housing and experimental tanks and provided diffuse illumination. The water level in the experimental arena was kept constant at 7 cm depth to minimize *D. rerio* motion across the water column and approximate movements along the horizontal plane.

At the beginning of each trial, a single *D. rerio* was acclimated for 5 min in a transparent plastic cylinder placed in the centre of the arena (acclimation period). A bird's eye webcam (C920 HD Pro, Logitech; http://www.logitech.com) recorded *D. rerio* movement for a total of 5 min after the cylinder had been carefully removed (first behavioural trial). Subsequently, each individual was transferred back into its individual tank for 30 min before repeating the measurement (second behavioural trial), as described above. After the finalization of the second behavioural trial, both *L*_T_ (to the nearest 0·1 mm) and body mass (to the nearest 0·01 g) of each individual were measured.

Videos were analysed using the video tracking software EthoVision XT Version 9.0 (Noldus Information Technologies Inc.; http://www.noldus.com), with position scoring starting 10 s after the cylinder had been removed. A total of 250 s were analysed for each individual per trial, with smoothing positions based on 10 samples. The space use of each *D. rerio* was calculated by virtually dividing the circular arena into three concentric zones: inner region (approximately six body‐lengths in diameter), middle region (approximately three body‐lengths in diameter wider than the inner region) and external region (approximately three body‐lengths in diameter wider than the middle region). Preliminary analyses, however, showed comparable scores for the time spent by *D. rerio* between the inner and middle region, while individuals were observed to spend most of the time within the three body‐lengths region away from the tank wall (*i.e*. external region). Thus, inner and middle regions were combined and two main zones of the arena were considered only: open‐water area (approximately nine body lengths from the centre of the arena) and proximity to the wall [approximately three body lengths from the wall of the arena, a measure consistent with Wright & Krause (2006) and Ladu *et al.* (2015)]. Distance moved (a proxy for activity and exploration and defined as total swimming distance per 250 s, measured in cm), time spent freezing (a proxy for inactivity and defined as time an individual was moving at speeds lower than 20 mm s^−1^ within any given 250 s, measured in s) and swimming in open water (a measure of risk‐taking and defined as time an individual was moving in the open‐water area at speeds higher than 20 mm s^−1^ within any given 250 s, measured in s) were extracted from the *xy*‐co‐ordinates obtained for each frame. Mean velocity was then calculated as distance moved per (250 s—time spent freezing). Notably, since freezing behaviour can be also associated with anxiogenic states in *D. rerio* and to antipredatory responses (Gerlai, 2010), the risk‐taking propensity of juvenile *D. rerio* was estimated here with respect to their time spent actively swimming in the open‐water area of arena, while the lack of mobility (*i.e*. freezing) was not included in the analysis.

### Statistical analysis

Prior to all analyses, distance moved was square‐root transformed and swimming in open water was log_10_‐transformed to achieve normal error distributions. Subsequently, dependent and continuous explanatory variables were centred on their grand mean following Dingemanse & Dochtermann (2013).

As distance moved, mean velocity, freezing and swimming in open water were measured synchronously, these behavioural traits may all represent components of the same personality axis [*i.e*. trait (Réale *et al.*, 2007; Carter *et al.*, 2013)]. Therefore, in a first step bi‐variate correlations between all behavioural traits were estimated through phenotypic correlations [*i.e*. the overall correlation jointly contributed by between‐ and within‐individual correlations (Dingemanse & Dochtermann, 2013)] by using bivariate linear mixed‐effects models (LMMs), as suggested by Dingemanse & Dochtermann (2013). The individual was specified as a random effect (*i.e*. random intercepts) to account for repeated measures of the same individual across trials.

To represent *D. rerio* condition, the Fulton's condition factor *K* (Pope & Kruse, 2007) was calculated for each individual. Although the correlation between Fulton's *K* (g mm^−3^ × 100 000) and *L*_T_ was not significant, the *P*‐value was low (Table I). To be conservative, a size‐controlled index of Fulton's *K* was derived by using the residuals of a regression of log_10_‐transformed *K* on log_10_‐transformed *L*_T_. Thus, residuals from Fulton's *K* were used for subsequent analysis serving as a condition index that was independent of length; it indicated the mass or nutritional state for a given individual relative to the standard size.

The presence of significant among‐individual differences in each behavioural trait was tested separately using LMMs (Dingemanse & Dochtermann, 2013) with individual random intercepts. Gape‐limited predators will be more constrained by prey length, as opposed to prey mass (Lorenzen, 2000). Thus, *L*_T_ (mm), residuals of (mass‐specific) RMR (controlled for *L*_T_), residuals of Fulton's *K* (controlled for *L*_T_) and trial were included as fixed factors, and the resulting among‐individual (intercept) and within‐individual (residual) variance estimates were used to calculate repeatability for each behavioural trait (Nakagawa & Schielzeth, 2010; Dingemanse & Dochtermann, 2013). Repeatability was defined as the proportion of the behavioural variation attributable to differences among individuals (Dingemanse & Dochtermann, 2013). As models included fixed factors, estimates of adjusted repeatability (Nakagawa & Schielzeth, 2010) are presented, *i.e*. the proportion of phenotypic variance not explained by fixed factors, which is attributable to consistent among‐individual differences in behaviour. Subsequently, the contribution of each fixed factor to the overall among‐individual variance in behaviour was investigated by including *L*_T_, residuals of RMR and residuals of Fulton's *K* independently in each LMM. Note that the fixed factor trial was consistently included in the ANOVA components as it does not represent a state variable (Sih *et al.*, 2015). Significance levels of fixed (*L*_T_, residuals RMR, residuals *K* and trial) and random factors were calculated using likelihood ratio tests.

The base model described above was inspired by the idea that *L*_T_ would exert a fundamental effect on activity and risk‐taking but that individuals that are in a poorer nutritional state or that carry higher‐than‐average mass‐specific metabolic demands would be more explorative and bolder. Models were therefore built with residuals of mass‐specific RMR controlling for *L*_T_. Alternative models are also possible, notably substituting absolute RMR for mass‐specific RMR and body mass as an index for *L*_T_. These alternative model variants were also tested, with residuals of log‐transformed absolute RMR (mg O_2_ h^−1^) controlling for *L*_T_ and using *L*_T_ as the fixed factor. Models were also built substituting body mass for *L*_T_ (because length and mass are highly correlated) taking residuals from both the log‐transformed absolute RMR and log‐transformed Fulton's *K* when regressed on log body mass. Results of these alternative models, that used measure of absolute rather than mass‐specific RMR and body mass rather than *L*_T_ as a morphological measure, entirely agreed with the model introduced in the previous paragraph. Alternative model variants are presented in Tables SI and SII, Supporting information.

The data analysis was performed in R 3.0.2 version (http://www.r-project.org). The bivariate LMMs were performed using MCMC sampling methods under a Bayesian framework [R package ‘MCMCglmm’; (Hadfield, 2010)]. The parameters were estimated using a non‐informative prior, with 1 500 000 resamplings, 500 000 burn‐ins and 100 thinnings. Other LMMs were performed with lme4 and nlme R packages (Pinheiro *et al.*, 2007; Bates *et al.*, 2014), respectively. The significance level was set at *P* ≤ 0·05. The s.e. of the mean is provided for each variance estimate in the results.

## Results

**Figure 1:**
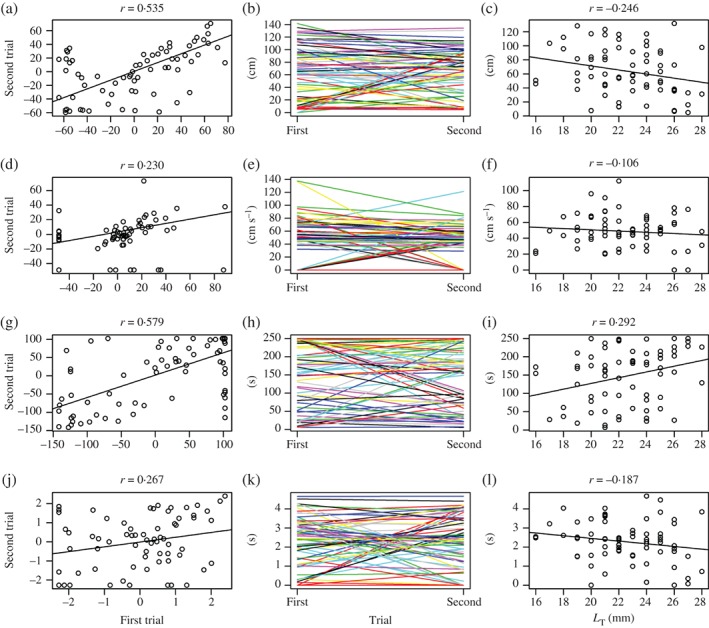
(a), (b), (d), (e), (g), (h), (j), (k) Behavioural repeatability and (c), (f), (i), (l) correlation of *Danio rerio* behaviour with total length (*L*_T_) are expressed with respect to (a)–(c) distance moved, (d)–(f) mean velocity, (g)–(i) freezing and (j)–(l) swimming in open water. Correlation coefficients (*r*) are given for each behavioural trait. Note co‐ordinates of each data point in (c), (f), (i), (l) represent the mean value calculated from the two behavioural measurements performed on each individual.

All four behavioural traits recorded in the open field were strongly correlated (Table II). LMMs detected significant effects, however, of the fixed factor *L*_T_ in relation to distance moved and freezing only. Accordingly, larger individuals swam on average shorter distances and spent more time freezing than smaller individuals of the same cohort (Table III and Fig. 1). Such effects of *L*_T_ on behaviour were not observed with respect to swimming in open water and mean velocity (Table III and Fig. 1). Nevertheless, the ANOVA yielded the highest *F*‐value, and the corresponding lowest *P*‐value, for the fixed factor *L*_T_ also in relation to swimming in open water, suggesting *L*_T_ to influence risk‐taking behaviour in *D. rerio*, albeit marginally, more than the other state variables measured in this study. There were no significant effects of mass‐specific RMR (residuals), Fulton's *K* (residuals) and trial on any of the four behavioural traits measured in the present study (Table III).

**Table II:** Phenotypic‐correlation estimates between pairs of behavioural traits. The best estimate of correlation coefficients (*i.e.* values above the diagonal) and their 95% c.i. (*i.e.* values below the diagonal) are represented for each pair of behavioural traits. Bivariate linear mixed‐effects models were used with Markov Chain Monte Carlo techniques, while including individuals as a random effect (*i.e.* random intercepts) to account for repeated measures. Significant results correspond to correlation coefficients whose 95% c.i. do not overlap with zero

	Distance moved	Mean velocity	Freezing	Swimming in open water
Distance moved		0·416	−0·944	0·533
Mean velocity	0·269		−0·245	−0·025
	0·551			
Freezing	−0·963	−0·402		−0·568
	−0·923	−0·081		
Swimming in open water	0·393	−0·055	−0·681	
	0·655	0·272	−0·437	

**Table III:** Results from the LMMs with distance moved, mean velocity, freezing and swimming in open water as dependent variables. Trial, total length (*L*_T_; mm) and *L*_T_‐corrected residuals from the log_10_‐transformed mass‐specific routine metabolic rate (RMR, mg O_2_ kg^−1^ h^−1^) and log_10_‐transformed Fulton's *K* (g mm^−3^ × 100 000) are included as fixed factors. Random intercepts are also included for each individual, which allowed variance decomposition. Intercepts (*V*_among_), residuals (*V*_within_) and adjusted repeatabilities are also shown with respect to each behavioural trait. Note that the fixed factor trial was consistently included for the ANOVA components: LMMs with one fixed factor per time (first three rows of the variance components) and LMM that includes all fixed factors (last row of the variance components)

Distance moved				
Fixed factors	Estimate	*F*	d.f._2_, d.f._1_	*P*
*L*_T_	−2·93	4·41	4, 5	<0·05
RMR	2·47	0·17	4, 5	>0·05
*K*	−17·43	0·22	4, 5	>0·05
Trial	−0·78	0·03	4, 5	>0·05
Random factor		*F*	d.f._2_, d.f._1_	*P*
Individual		—	6, 7	<0·001
Variance components		*V*_within_ ± s.e.	*V*_among_ ± s.e.	Repeatability
*L*_T_		710·9 ± 2·3	720·6 ± 2·3	0·50
RMR		710·9 ± 2·3	787·0 ± 2·4	0·53
*K*		710·9 ± 2·3	785·2 ± 2·4	0·53
*L*_T_, RMR, *K*		710·9 ± 2·3	714·3 ± 2·3	**0·50**
**Mean velocity**				
Fixed factors	Estimate	*F*	d.f._2_, d.f._1_	*P*
*L*_T_	−0·78	0·76	4, 5	>0·05
RMR	6·01	0·92	4, 5	>0·05
*K*	20·88	0·87	4, 5	>0·05
Trial	−5·17	1·58	4, 5	>0·05
Random factor		*F*	d.f._2_, d.f._1_	*P*
Individual		—	6, 7	0·05
Variance components		*V*_within_ ± s.e.	*V*_among_ ± s.e.	Repeatability
*L*_T_		575·6 ± 2·1	160·7 ± 1·1	0·22
RMR		575·6 ± 2·1	159·8 ± 1·1	0·22
*K*		575·6 ± 2·1	162·4 ± 1·1	0·22
*L*_T_, RMR, *K*		575·6 ± 2·1	149·4 ± 1·1	**0·21**
**Freezing**				
Fixed factors	Estimate	*F*	d.f._2_, d.f._1_	*P*
*L*_T_	7·85	6·45	4, 5	<0·05
RMR	−5·59	0·24	4, 5	>0·05
*K*	64·34	0·62	4, 5	>0·05
Trial	−2·67	0·08	4, 5	>0·05
Random factor		*F*	d.f._2_, d.f._1_	*P*
Individual		—	6, 7	<0·001
Variance components		*V*_within_ ± s.e.	*V*_among_ ± s.e.	Repeatability
*L*_T_		3112 ± 5	3770 ± 5	0·55
RMR		3112 ± 5	4248 ± 6	0·58
*K*		3112 ± 5	4209 ± 6	0·57
*L*_T_, RMR, *K*		3112 ± 5	3701 ± 5	**0·54**
**Swimming in open water**				
Fixed factors	Estimate	*F*	d.f._2_, d.f._1_	*P*
*L*_T_	−0·07	2·59	4, 5	>0·05
RMR	−0·09	<0·01	4, 5	>0·05
*K*	−1·57	1·91	4, 5	>0·05
Trial	<0·01	<0·01	4, 5	>0·05
Random factor		*F*	d.f._2_, d.f._1_	*P*
Individual		—	6, 7	<0·05
Variance components		*V*_within_ ± s.e.	*V*_among_ ± s.e.	Repeatability
*L*_T_		1·32 ± 0·10	0·44 ± 0·06	0·25
RMR		1·32 ± 0·10	0·48 ± 0·06	0·27
*K*		1·32 ± 0·10	0·45 ± 0·06	0·25
*L*_T_, RMR, *K*		1·32 ± 0·10	0·41 ± 0·05	**0·24**

After accounting for *L*_T_ and other fixed factors, significant among‐individual variation in each behavioural trait remained, yielding repeatability estimates which comprised between 0·21 and 0·54 (Table III and Fig. 1). In other words, even after controlling for *L*_T_ effects, consistent variation in *D. rerio* behaviour in the open field was present among similar sized individuals. Further analyses using LMMs including all fixed factors revealed that among‐individual differences for each behavioural trait were significantly affected by the random effect (individual ID; Table III), confirming the presence of personality (or behavioural types) in juvenile *D. rerio*.

## Discussion

In the present study, smaller juvenile *D. rerio* exhibited consistently higher activity (*i.e*. higher distance moved and lower freezing) in a novel and potentially dangerous open field compared to larger conspecifics of the same cohort, while swimming in open water (*i.e*. a direct measure of risk‐taking) was only marginally affected by individual size. On the contrary, no effects of either RMR or nutritional state were found on the propensity to explore and take risks in an open field. The present study thus supports the hypothesis that body length (*i.e. L*_T_) *per se* has a stronger effect than metabolism on activity and risk‐taking behaviours and in fact suggests that no relationship among RMR and these behaviours may exist in juvenile *D. rerio*, at least under the conditions examined here.

The study's key finding of a lack of relationship of size‐independent RMR and tendency to explore and take risks in *D. rerio* is contingent on the presence of personality in this species. The experimental design, however, has the limitation of a short time interval (30 min) between the two behavioural trials. It is unknown whether the fairly robust consistency in activity and risk‐taking reported here would persist over longer time periods (*e.g*. weeks or months). There is a large body of literature on *D. rerio* personality, however, all finding evidence of consistent individual variation in a range of personality traits, such as activity, risk‐taking, aggression and sociability (Moretz *et al.*, 2007; Dahlbom *et al.*, 2011; Ariyomo & Watt, 2012; Tran & Gerlai, 2013). It is thus believed that personality is present in *D. rerio*, which adds weight to the conclusion that routine metabolism may play only a limited role, or no role at all, in determining consistent variation in activity and risk‐taking in this species when body length (*i.e. L*_T_) is accounted for.

In the present study, the *L*_T_ of juvenile *D. rerio* was found to be a strong predictor of activity, which is strongly associated with boldness in *D. rerio* (Moretz *et al.*, 2007), but only marginally linked to a more direct measure of risk‐taking behaviour in individuals from the same cohort. In particular, smaller individuals were found to be more active and risk prone in a novel, shelterless and potentially dangerous environment, which is in agreement with earlier studies on other fish species (Krause *et al.*, 1998; Brown & Braithwaite, 2004; Biro *et al.*, 2005). Size‐dependent phenomena are well known in ecology and size scaling relationships have been well described in fishes (Schmidt‐Nielsen, 1984; Clarke & Johnston, 1999; Killen *et al.*, 2010; White & Kearney, 2014). Indeed, small fishes of a given cohort typically possess lower energy reserves and higher mass‐specific metabolic demands, which is believed to generally favour elevated propensities to explore new environments and take risks because smaller individuals should have more immediate needs for finding food (Wootton, 1994; Krause *et al.*, 1998). Following this argument, Krause *et al.* (1998) observed that small three‐spined sticklebacks *Gasterosteus aculeatus* L. 1758 were consistently faster in emerging from a refuge and spent more time exploring a novel environment compared to larger conspecifics. These smaller *G. aculeatus* also showed larger mass loss when deprived of food, motivating the explanation that the increased activity and risk‐taking of small fishes may be associated with metabolic demands (Krause *et al.*, 1998). Here, a metabolism effect in *D. rerio* that differed in size was controlled for, in turn rendering the metabolic explanation for the size‐effect on activity and boldness unlikely. Instead, the present work may suggest that smaller *D. rerio* of a given cohort were outcompeted for food in their holding tanks, in turn motivating elevated exploratory and risk‐taking tendencies in these starved individuals when observed in isolation. Effects of nutritional states on activity and marginally on risk‐taking, however, were only present in relation to using *L*_T_ as fixed factor, with no further role related to body condition. *L*_T_ variation in juvenile *D. rerio* might more readily indicate variation of nutritional states than variation in Fulton' *K*. Indeed, findings from the present study do not support an effect of Fulton's *K* in shaping different risk strategies in *D. rerio* and other works have also raised cautionary notes about the index values of Fulton's *K* to properly capture nutritional variation in fishes (Morton & Routledge, 2006; Klefoth *et al.*, 2013). This explanation might help understanding the lack of predictive power of body condition in the present work.

A second evolutionary mechanism may have contributed to the present study's findings in relation to the correlation between *L*_T_ and activity and boldness. In contrast to most species (Lorenzen, 2000), larger size classes may be under greater risk of predation in small‐bodied fish species because they offer greater energy packs for predators compared to smaller conspecifics (Persson *et al.*, 2003; Johansson *et al.*, 2004). Hence, larger fish of small‐bodied species, in which large size does not necessarily confer a mortality advantage, may reduce their propensity to take risks as an evolutionary adaptation to predation risk in their natural environment. Such adaptation would in turn show up as greater activity and boldness of smaller fishes in laboratory tests. Fishes have indeed developed a wide array of strategies in the juvenile stage aimed at trading‐off key fitness components, particularly growth and mortality (Werner & Hall, 1988; Werner & Anholt, 1993; Clark, 1994; Sogard, 1997; Metcalfe & Monaghan, 2001; Biro *et al.*, 2005; Dial *et al.*, 2008). One of these strategies is feeding in the presence of risk rather than waiting for safer feeding opportunities, which can lead to starvation and possibly mortality in the long term (Werner & Hall, 1988; Werner & Anholt, 1993; Clark, 1994; Biro *et al.*, 2005). In this context, the risk‐taking behaviour of juvenile rainbow trout *Oncorhynchus mykiss* (Walbaum 1792) was found to decrease with time in favour of exploration as a function of size, *i.e*. only small individuals were highly motivated to explore the environment and feed under risk of predation to increase growth and minimize the time spent in vulnerable sizes (Biro *et al.*, 2005). Moreover, small guppies *Poecilia reticulata* Peters 1859 have been found to be more exploratory and successful in discovering novel food sources in a novel and potentially dangerous environment compared to larger conspecifics (Laland & Reader, 1999). Similar results were found in other small‐bodied poeciliids (Brown & Braithwaite, 2004), where smaller individuals emerged consistently sooner from a sheltered area compared to larger ones, probably in an attempt to balance predation risk with foraging rate and energy gain. An evolutionary adaptation to reduce predation risk of large‐bodied animals, however, cannot be ruled out. Similarly, it is possible that the individuals used in the present study were evolutionarily adapted to the predators in their home habitat, *e.g*. the Indian leaf fish *Nandus nandus* (Hamilton 1822) (Bass & Gerlai, 2008). Therefore, the presented finding that smaller juvenile *D. rerio* were more active and, thus, more risk‐prone than larger ones aligns with two non‐mutually exclusive explanations: either these behaviours are shown to permit the smallest size classes of a given cohort to safeguard access to food in order to grow and reach sizes that substantially reduce the probability of predatory attacks, or the pattern can be explained by evolutionary adaptation to combat greater predation risk in large size classes by becoming inactive and shyer as the individual's length increases. Only ontogenetic studies where individuals are repeatedly assayed in high and low food situations can disentangle these competing hypotheses.

The present results did not support the expected covariance between RMR and personality in *D. rerio*, which agrees with previous works reporting inconsistent associations between metabolic rates and behavioural types in a range of animals (Seppänen *et al.*, 2009; Vaz‐Serrano *et al.*, 2011; Bouwhuis *et al.*, 2014; Guenther *et al.*, 2014; Killen *et al.*, 2014). In European sea bass *Dicentrarchus labrax* (L. 1758) the correlation between metabolic rate and spontaneous swimming activity, as an index of risk‐taking behaviour, was found to vary across environmental contexts (Killen *et al.*, 2012). After a predator attack, individuals with higher RMR tended to emerge sooner from a sheltered area and were more active than their conspecifics with lower metabolic demands only under hypoxia, while no relationship between activity or risk‐taking and metabolic rate was observed when oxygen availability was high (Killen *et al.*, 2012). Killen *et al.* (2013) have recently suggested that the presence or intensity of environmental stressors can modulate covariation between metabolic traits and animal personality. In particular, moderate levels of food deprivation or risk of predation may persuade individuals with high energetic demands to become more active or prone to take risks as they attempt to find food, thus pronouncing the latent links between metabolic traits, activity and risk‐taking (Killen *et al.*, 2013). A corollary of the argument is that relationships between metabolism and behavioural phenotypes might not be manifested under relatively benign conditions (Killen *et al.*, 2013). Although the open field test is assumed to be perceived as a threat by experimental animals, this stress alone may not have been sufficient to reveal ecologically relevant variation in behaviours among *D. rerio* stemming from variation in RMR. In fact, as is common in laboratory studies, water quality variables adopted here were optimal for *D. rerio* (Avdesh *et al.*, 2011). Focal individuals were also free from starvation and actual risk of predation since no real predators were involved in the experimental design, while age at the time of testing was selected to prevent the manifestation of aggressive and territorial tendencies among *D. rerio* that are typical at reproductive maturity (Spence *et al.*, 2008). Therefore, in the absence of strong environmental stressors that may challenge individuals and require them to adjust behaviour to fit their intrinsic variation in RMR, fluctuations in behavioural and metabolic traits of juvenile *D. rerio* may have acted independently and hence the two phenotypes were uncorrelated. Nevertheless, the data suggest that even if there is a relationship of metabolism and behaviour in different environmental contexts, the relationship is subtle and may be overridden by state effects related to body size. Note that the present work used *D. rerio* of the same cohort and hence variation in *L*_T_ was controlled to a small length range, such that effects of *L*_T_ on activity and risk‐taking may be more pronounced if a greater length range is examined.

In conclusion, activity and in part risk‐taking in juvenile *D. rerio* were found to vary as a function of individual *L*_T_, while there was little evidence for correlations between these behaviours and length‐independent RMR or body condition. Future studies shall explore behavioural and metabolic responses along ontogenetic transitions of *D. rerio* (or other species) and in different environments to uncover the relative role of state and physiological underpinning in shaping behavioural variation and personality.

The authors would like to gratefully acknowledge K. Laskowski and F. Pilotto for their valuable help on the statistical analysis and T. Mehner and the participants of the workshop ‘Scientific Writing’ at the Leibniz‐Institute of Freshwater Ecology and Inland Fisheries (IGB) for helpful discussions on an early stage of the manuscript. The authors would also like to thank the two anonymous reviewers for the useful suggestions that have helped to improve the presentation of this manuscript. S.S.K. was supported by a NERC Advanced Fellowship NE/J019100/1. This work was part of the B‐Types project (http://www.b-types.igb-berlin.de/) funded through the Leibniz Competition (SAW‐2013‐IGB‐2) and was also funded by the Adaptfish grant funded through the Leibniz‐Competition to R.A. The experiments described here were approved under HO Project License 60/4461.

## Supporting information


**table SI.** Results from the LMMs with distance moved, mean velocity, freezing and swimming in open water as dependent variables. Trial, *L*_T_ (mm) and *L*_T_‐corrected residuals from the log‐transformed absolute RMR (mg O_2_ h^−1^) and log‐transformed Fulton's *K* (g mm^−3^ × 100 000) are included as fixed factors. Random intercepts are also included for each individual, which allowed variance decomposition. Intercepts (*V*_among_), residuals (*V*_within_) and adjusted repeatabilities are also shown with respect to each behavioural trait. Note that the fixed factor trial was consistently included for the analysis of the variance components: LMMs with one fixed factor per time (first three rows of the variance components) and LMM that includes all fixed factors (last row of the variance components)
**table SII.** Results from the LMMs with distance moved, mean velocity, freezing and swimming in open water as dependent variables. Trial, body mass (g) and mass‐corrected residuals from the log‐transformed absolute RMR (mg O_2_ h^−1^) and log‐transformed Fulton's *K* (g mm^−3^ × 100 000) are included as fixed factors. Random intercepts are also included for each individual, which allowed variance decomposition. Intercepts (*V*_among_), residuals (*V*_within_) and adjusted repeatabilities are also shown with respect to each behavioural trait. Note that the fixed factor trial was consistently included for the analysis of the variance components: LMMs with one fixed factor per time (first three rows of the variance components) and LMM that includes all fixed factors (last row of the variance components)

## References

[jfb13100-bib-0001] Ahrens, R. N. , Walters, C. J. & Christensen, V. (2012). Foraging arena theory. Fish and Fisheries 13, 41–59.

[jfb13100-bib-0002] Ariyomo, T. O. & Watt, P. J. (2012). The effect of variation in boldness and aggressiveness on the reproductive success of zebrafish. Animal Behaviour 83, 41–46.

[jfb13100-bib-0003] Avdesh, A. , Chen, M. , Martin‐Iverson, M. T. , Mondal, A. , Ong, D. , Rainey‐Smith, S. , Taddei, K. , Lardelli, M. , Groth, D. M. , Verdile, G. & Martins, R. N. (2011). Regular care and maintenance of a zebrafish (*Danio rerio*) laboratory: an introduction. Journal of Visualized Experiments 69, e4196. 10.3791/4196 PMC3916945 23183629

[jfb13100-bib-0004] Bass, S. L. & Gerlai, R. (2008). Zebrafish (*Danio rerio*) responds differentially to stimulus fish: the effects of sympatric and allopatric predators and harmless fish. Behavioural Brain Research 186, 107–117. 17854920 10.1016/j.bbr.2007.07.037

[jfb13100-bib-0005] Biro, P. A. & Stamps, J. A. (2008). Are animal personality traits linked to life‐history productivity? Trends in Ecology & Evolution 23, 361–368. 18501468 10.1016/j.tree.2008.04.003

[jfb13100-bib-0006] Biro, P. A. & Stamps, J. A. (2010). Do consistent individual differences in metabolic rate promote consistent individual differences in behavior? Trends in Ecology & Evolution 25, 653–659. 20832898 10.1016/j.tree.2010.08.003

[jfb13100-bib-0007] Biro, P. A. , Post, J. R. & Abrahams, M. V. (2005). Ontogeny of energy allocation reveals selective pressure promoting risk‐taking behaviour in young fish cohorts. Proceedings of the Royal Society B 272, 1443–1448. 16011918 10.1098/rspb.2005.3096 PMC1559824

[jfb13100-bib-0008] Bouwhuis, S. , Quinn, J. L. , Sheldon, B. C. & Verhulst, S. (2014). Personality and basal metabolic rate in a wild bird population. Oikos 123, 56–62.

[jfb13100-bib-0009] Brown, C. & Braithwaite, V. A. (2004). Size matters: a test of boldness in eight populations of the poeciliid *Brachyraphis episcopi* . Animal Behaviour 68, 1325–1329.

[jfb13100-bib-0010] Brown, C. , Jones, F. & Braithwaite, V. A. (2007). Correlation between boldness and body mass in natural populations of the poeciliid *Brachyrhaphis episcopi* . Journal of Fish Biology 71, 1590–1601.

[jfb13100-bib-0011] Burns, J. G. (2008). The validity of three tests of temperament in guppies (*Poecilia reticulata*). Journal of Comparative Psychology 122, 344. 19014258 10.1037/0735-7036.122.4.344

[jfb13100-bib-0012] Buskirk, J. V. (2012). Behavioural plasticity and environmental change In Behavioural Responses to a Changing World: Mechanisms and Consequences (Candolin U. & Wong B. B. M., eds), pp. 145–158. Oxford: Oxford University Press.

[jfb13100-bib-0013] Cahill, G. M. (2002). Clock mechanisms in zebrafish. Cell and Tissue Research 309, 27–34. 12111534 10.1007/s00441-002-0570-7

[jfb13100-bib-0014] Carere, C. & Maestripieri, D. (2013). Animal Personalities: Behavior, Physiology, and Evolution. Chicago, IL: University of Chicago Press.

[jfb13100-bib-0015] Carter, A. J. , Feeney, W. E. , Marshall, H. H. , Cowlishaw, G. & Heinsohn, R. (2013). Animal personality: what are behavioural ecologists measuring? Biological Reviews 88, 465–475. 23253069 10.1111/brv.12007

[jfb13100-bib-0016] Clark, C. W. (1994). Antipredator behavior and the asset‐protection principle. Behavioral Ecology 5, 159–170.

[jfb13100-bib-0017] Clarke, A. & Johnston, N. M. (1999). Scaling of metabolic rate with body mass and temperature in teleost fish. Journal of Animal Ecology 68, 893–905.

[jfb13100-bib-0018] Dahlbom, S. J. , Lagman, D. , Lundstedt‐Enkel, K. , Sundström, L. F. & Winberg, S. (2011). Boldness predicts social status in zebrafish (*Danio rerio*). PLoS One 6, e23565. 21858168 10.1371/journal.pone.0023565 PMC3157393

[jfb13100-bib-0019] Dial, K. P. , Greene, E. & Irschick, D. J. (2008). Allometry of behavior. Trends in Ecology & Evolution 23, 394–401. 18501467 10.1016/j.tree.2008.03.005

[jfb13100-bib-0020] Dingemanse, N. J. & Dochtermann, N. A. (2013). Quantifying individual variation in behaviour: mixed‐effect modelling approaches. Journal of Animal Ecology 82, 39–54. 23171297 10.1111/1365-2656.12013

[jfb13100-bib-0021] Dupont‐Prinet, A. , Chatain, B. , Grima, L. , Vandeputte, M. , Claireaux, G. & McKenzie, D. J. (2010). Physiological mechanisms underlying a trade‐off between growth rate and tolerance of feed deprivation in the European sea bass (*Dicentrarchus labrax*). The Journal of Experimental Biology 213, 1143–1152. 20228351 10.1242/jeb.037812

[jfb13100-bib-0022] Dwyer, G. K. , Stoffels, R. J. & Pridmore, P. A. (2014). Morphology, metabolism and behaviour: responses of three fishes with different lifestyles to acute hypoxia. Freshwater Biology 59, 819–831.

[jfb13100-bib-0023] Filby, A. L. , Paull, G. C. , Bartlett, E. J. , Van Look, K. J. & Tyler, C. R. (2010). Physiological and health consequences of social status in zebrafish (*Danio rerio*). Physiology & Behavior 101, 576–587. 20851709 10.1016/j.physbeh.2010.09.004

[jfb13100-bib-0024] Gerlai, R. (2010). Zebrafish antipredatory responses: a future for translational research? Behavioural Brain Research 207, 223–231. 19836422 10.1016/j.bbr.2009.10.008 PMC3203216

[jfb13100-bib-0025] Grant, J. W. (1990). Aggressiveness and the foraging behaviour of young‐of‐the‐year brook charr (*Salvelinus fontinalis*). Canadian Journal of Fisheries and Aquatic Sciences 47, 915–920.

[jfb13100-bib-0026] Guenther, A. & Trillmich, F. (2015). Within‐litter differences in personality and physiology relate to size differences among siblings in cavies. Physiology & Behavior 145, 22–28. 25802020 10.1016/j.physbeh.2015.03.026

[jfb13100-bib-0027] Guenther, A. , Finkemeier, M. A. & Trillmich, F. (2014). The ontogeny of personality in the wild guinea pig. Animal Behaviour 90, 131–139.

[jfb13100-bib-0028] Hadfield, J. D. (2010). MCMC methods for multi‐response generalized linear mixed models: the MCMCglmm R package. Journal of Statistical Software 33, 1–22. 20808728 PMC2929880

[jfb13100-bib-0029] Healy, K. , McNally, L. , Ruxton, G. D. , Cooper, N. & Jackson, A. L. (2013). Metabolic rate and body size are linked with perception of temporal information. Animal Behaviour 86, 685–696. 24109147 10.1016/j.anbehav.2013.06.018 PMC3791410

[jfb13100-bib-0030] Johansson, J. , Turesson, H. & Persson, A. (2004). Active selection for large guppies, *Poecilia reticulata*, by the pike cichlid, *Crenicichla saxatilis* . Oikos 105, 595–605.

[jfb13100-bib-0031] Johnsson, J. I. (1993). Big and brave: size selection affects foraging under risk of predation in juvenile rainbow trout, *Oncorhynchus mykiss* . Animal Behaviour 45, 1219–1225.

[jfb13100-bib-0032] Killen, S. S. , Atkinson, D. & Glazier, D. S. (2010). The intraspecific scaling of metabolic rate with body mass in fishes depends on lifestyle and temperature. Ecology Letters 13, 184–193. 20059525 10.1111/j.1461-0248.2009.01415.x

[jfb13100-bib-0033] Killen, S. S. , Marras, S. & McKenzie, D. J. (2011). Fuel, fasting, fear: routine metabolic rate and food deprivation exert synergistic effects on risk‐taking in individual juvenile European sea bass. Journal of Animal Ecology 80, 1024–1033. 21790592 10.1111/j.1365-2656.2011.01844.x

[jfb13100-bib-0034] Killen, S. S. , Marras, S. , Ryan, M. R. , Domenici, P. & McKenzie, D. J. (2012). A relationship between metabolic rate and risk‐taking behaviour is revealed during hypoxia in juvenile European sea bass. Functional Ecology 26, 134–143.

[jfb13100-bib-0035] Killen, S. S. , Marras, S. , Metcalfe, N. B. , McKenzie, D. J. & Domenici, P. (2013). Environmental stressors alter relationships between physiology and behaviour. Trends in Ecology & Evolution 28, 651–658. 23756106 10.1016/j.tree.2013.05.005

[jfb13100-bib-0036] Killen, S. S. , Mitchell, M. D. , Rummer, J. L. , Chivers, D. P. , Ferrari, M. C. , Meekan, M. G. & McCormick, M. I. (2014). Aerobic scope predicts dominance during early life in a tropical damselfish. Functional Ecology 28, 1367–1376.

[jfb13100-bib-0037] Klefoth, T. , Skov, C. , Aarestrup, K. & Arlinghaus, R. (2013). Reliability of non‐lethal assessment methods of body composition and energetic status exemplified by applications to eel (*Anguilla anguilla*) and carp (*Cyprinus carpio*). Fisheries Research 146, 18–26.

[jfb13100-bib-0038] Kobler, A. , Klefoth, T. , Wolter, C. , Fredrich, F. & Arlinghaus, R. (2008). Contrasting pike (*Esox lucius* L.) movement and habitat choice between summer and winter in a small lake. Hydrobiologia 601, 17–27.

[jfb13100-bib-0039] Kopman, V. , Laut, J. , Polverino, G. & Porfiri, M. (2013). Closed‐loop control of zebrafish response using a bioinspired robotic‐fish in a preference test. Journal of the Royal Society Interface 10, 20120540. 10.1098/rsif.2012.0540 PMC3565779 23152102

[jfb13100-bib-0040] Krause, J. , Loader, S. P. , McDermott, J. & Ruxton, G. D. (1998). Refuge use by fish as a function of body length‐related metabolic expenditure and predation risks. Proceedings of the Royal Society of London B 265, 2373–2379.

[jfb13100-bib-0041] Ladu, F. , Bartolini, T. , Panitz, S. G. , Chiarotti, F. , Butail, S. , Macrì, S. & Porfiri, M. (2015). Live predators, robots, and computer‐animated images elicit differential avoidance responses in zebrafish. Zebrafish 12, 205–214. 25734228 10.1089/zeb.2014.1041

[jfb13100-bib-0042] Laland, K. N. & Reader, S. M. (1999). Foraging innovation in the guppy. Animal Behaviour 57, 331–340. 10049472 10.1006/anbe.1998.0967

[jfb13100-bib-0043] Landsman, S. J. , Martins, E. G. , Gutowsky, L. F. , Suski, C. D. , Arlinghaus, R. & Cooke, S. J. (2015). Locomotor activity patterns of muskellunge (*Esox masquinongy*) assessed using tri‐axial acceleration sensing acoustic transmitters. Environmental Biology of Fishes 98, 2109–2121.

[jfb13100-bib-0044] Lorenzen, K. (2000). Allometry of natural mortality as a basis for assessing optimal release size in fish‐stocking programmes. Canadian Journal of Fisheries and Aquatic Sciences 57, 2374–2381.

[jfb13100-bib-0045] Lucas, J. , Schouman, A. , Lyphout, L. , Cousin, X. & Lefrancois, C. (2014). Allometric relationship between body mass and aerobic metabolism in zebrafish *Danio rerio* . Journal of Fish Biology 84, 1171–1178. 24628562 10.1111/jfb.12306

[jfb13100-bib-0046] Mathot, K. J. & Dingemanse, N. J. (2015). Energetics and behavior: unrequited needs and new directions. Trends in Ecology & Evolution 30, 199–206. 25687159 10.1016/j.tree.2015.01.010

[jfb13100-bib-0047] Metcalfe, N. B. & Monaghan, P. (2001). Compensation for a bad start: grow now, pay later? Trends in Ecology & Evolution 16, 254–260. 11301155 10.1016/s0169-5347(01)02124-3

[jfb13100-bib-0048] Metcalfe, N. B. , Van Leeuwen, T. E. & Killen, S. S. (2016). Does individual variation in metabolic phenotype predict behaviour and performance in fish? Journal of Fish Biology 88, 298–321. 26577442 10.1111/jfb.12699 PMC4991269

[jfb13100-bib-0049] Moretz, J. A. , Martins, E. P. & Robison, B. D. (2007). Behavioral syndromes and the evolution of correlated behavior in zebrafish. Behavioral Ecology 18, 556–562.

[jfb13100-bib-0050] Morton, A. & Routledge, R. D. (2006). Fulton's condition factor: is it a valid measure of sea lice impact on juvenile salmon? North American Journal of Fisheries Management 26, 56–62.

[jfb13100-bib-0051] Nakagawa, S. & Schielzeth, H. (2010). Repeatability for Gaussian and non‐Gaussian data: a practical guide for biologists. Biological Reviews 85, 935–956. 20569253 10.1111/j.1469-185X.2010.00141.x

[jfb13100-bib-0052] Nilsson, P. A. & Brönmark, C. (2000). Prey vulnerability to a gape‐size limited predator: behavioural and morphological impacts on northern pike piscivory. Oikos 88, 539–546.

[jfb13100-bib-0053] Nussey, D. H. , Wilson, A. J. & Brommer, J. E. (2007). The evolutionary ecology of individual phenotypic plasticity in wild populations. Journal of Evolutionary Biology 20, 831–844. 17465894 10.1111/j.1420-9101.2007.01300.x

[jfb13100-bib-0054] Oikawa, S. , Itazawa, Y. & Gotoh, M. (1991). Ontogenetic change in the relationship between metabolic rate and body mass in a sea bream *Pagrus major* (Temminck & Schlegel). Journal of Fish Biology 38, 483–496.

[jfb13100-bib-0055] Persson, L. , De Roos, A. M. , Claessen, D. , Byström, P. , Lövgren, J. , Sjögren, S. , Svanbäck, R. , Wahlström, E. & Westman, E. (2003). Gigantic cannibals driving a whole‐lake trophic cascade. Proceedings of the National Academy of Sciences 100, 4035–4039. 10.1073/pnas.0636404100 PMC153043 12646706

[jfb13100-bib-0056] Polverino, G. , Abaid, N. , Kopman, V. , Macrì, S. & Porfiri, M. (2012). Zebrafish response to robotic fish: preference experiments on isolated individuals and small shoals. Bioinspiration & Biomimetics 7, 036019. 22677608 10.1088/1748-3182/7/3/036019

[jfb13100-bib-0057] Polverino, G. , Ruberto, T. , Staaks, G. & Mehner, T. (2016). Tank size alters mean behaviours and individual rank orders in personality traits of fish depending on their life stage. Animal Behaviour 115, 127–135.

[jfb13100-bib-0058] Pope, K. L. & Kruse, C. G. (2007). Condition In Analysis and Interpretation of Freshwater Fisheries Data (Guy C. S. & Brown M. L., eds), pp. 423–471. Maryland, MD: American Fisheries Society.

[jfb13100-bib-0059] Pruitt, J. N. & Riechert, S. E. (2011). How within‐group behavioural variation and task efficiency enhance fitness in a social group. Proceedings of the Royal Society B 278, 1209–1215. 20943687 10.1098/rspb.2010.1700 PMC3049074

[jfb13100-bib-0060] Réale, D. , Reader, S. M. , Sol, D. , McDougall, P. T. & Dingemanse, N. J. (2007). Integrating animal temperament within ecology and evolution. Biological Reviews 82, 291–318. 17437562 10.1111/j.1469-185X.2007.00010.x

[jfb13100-bib-0061] Réale, D. , Garant, D. , Humphries, M. M. , Bergeron, P. , Careau, V. & Montiglio, P. O. (2010). Personality and the emergence of the pace‐of‐life syndrome concept at the population level. Philosophical Transactions of the Royal Society, B: Biological Sciences 365, 4051–4063. 10.1098/rstb.2010.0208 PMC2992747 21078657

[jfb13100-bib-0062] Schmidt‐Nielsen, K. (1984). Scaling: Why Is Animal Size so Important? Cambridge: Cambridge University Press.

[jfb13100-bib-0063] Schurmann, H. & Steffensen, J. F. (1997). Effects of temperature, hypoxia and activity on the metabolism of juvenile Atlantic cod. Journal of Fish Biology 50, 1166–1180.

[jfb13100-bib-0064] Seeman, T. E. & McEwen, B. S. (1996). Impact of social environment characteristics on neuroendocrine regulation. Psychosomatic Medicine 58, 459–471. 8902897 10.1097/00006842-199609000-00008

[jfb13100-bib-0065] Seppänen, E. , Tiira, K. , Huuskonen, H. & Piironen, J. (2009). Metabolic rate, growth and aggressiveness in three Atlantic salmon *Salmo salar* populations. Journal of Fish Biology 74, 562–575. 20735579 10.1111/j.1095-8649.2008.02142.x

[jfb13100-bib-0066] Sih, A. , Mathot, K. J. , Moiron, M. , Montiglio, P. O. , Wolf, M. & Dingemanse, N. J. (2015). Animal personality and state‐behaviour feedbacks: a review and guide for empiricists. Trends in Ecology & Evolution 30, 50–60. 25498413 10.1016/j.tree.2014.11.004

[jfb13100-bib-0067] Sogard, S. M. (1997). Size‐selective mortality in the juvenile stage of teleost fishes: a review. Bulletin of Marine Science 60, 1129–1157.

[jfb13100-bib-0068] Spence, R. , Gerlach, G. , Lawrence, C. & Smith, C. (2008). The behaviour and ecology of the zebrafish, *Danio rerio* . Biological Reviews 83, 13–34. 18093234 10.1111/j.1469-185X.2007.00030.x

[jfb13100-bib-0069] Steffensen, J. F. (1989). Some errors in respirometry of aquatic breathers: how to avoid and correct for them. Fish Physiology and Biochemistry 6, 49–59. 24226899 10.1007/BF02995809

[jfb13100-bib-0070] Tran, S. & Gerlai, R. (2013). Individual differences in activity levels in zebrafish (*Danio rerio*). Behavioural Brain Research 257, 224–229. 24084583 10.1016/j.bbr.2013.09.040 PMC4217171

[jfb13100-bib-0071] Uchmanski, J. (1985). Differentiation and frequency distributions of body weights in plants and animals. Philosophical Transactions of the Royal Society B 310, 1–75. 10.1098/rstb.1985.0099 2864710

[jfb13100-bib-0072] Vaz‐Serrano, J. , Ruiz‐Gomez, M. L. , Gjøen, H. M. , Skov, P. V. , Huntingford, F. A. , Øverli, Ø. & Höglund, E. (2011). Consistent boldness behaviour in early emerging fry of domesticated Atlantic salmon (*Salmo salar*): decoupling of behavioural and physiological traits of the proactive stress coping style. Physiology & Behavior 103, 359–364. 21352840 10.1016/j.physbeh.2011.02.025

[jfb13100-bib-0073] Werner, E. E. & Anholt, B. R. (1993). Ecological consequences of the trade‐off between growth and mortality rates mediated by foraging activity. American Naturalist 142, 242–272. 10.1086/285537 19425978

[jfb13100-bib-0074] Werner, E. E. & Hall, D. J. (1988). Ontogenetic habitat shifts in bluegill: the foraging rate‐predation risk trade‐off. Ecology 69, 1352–1366.

[jfb13100-bib-0075] White, C. R. & Kearney, M. R. (2013). Determinants of inter‐specific variation in basal metabolic rate. Journal of Comparative Physiology B 183, 1–26. 10.1007/s00360-012-0676-5 23001691

[jfb13100-bib-0076] White, C. R. & Kearney, M. R. (2014). Metabolic scaling in animals: methods, empirical results, and theoretical explanations. Comprehensive Physiology 4, 231–256. 24692144 10.1002/cphy.c110049

[jfb13100-bib-0077] Whiteley, A. R. , Bhat, A. , Martins, E. P. , Mayden, R. L. , Arunachalam, M. , Uusi‐Heikkilä, S. , Ahmed, A. T. A. , Shrestha, J. , Clark, M. , Stemple, D. & Bernatchez, L. (2011). Population genomics of wild and laboratory zebrafish (*Danio rerio*). Molecular Ecology 20, 4259–4276. 21923777 10.1111/j.1365-294X.2011.05272.x PMC3627301

[jfb13100-bib-0078] Wolf, M. & Weissing, F. J. (2010). An explanatory framework for adaptive personality differences. Philosophical Transactions of the Royal Society B 365, 3959–3968. 10.1098/rstb.2010.0215 PMC2992748 21078648

[jfb13100-bib-0079] Wolf, M. , van Doorn, G. S. , Leimar, O. & Weissing, F. J. (2007). Life‐history trade‐offs favour the evolution of animal personalities. Nature 447, 581–584. 17538618 10.1038/nature05835

[jfb13100-bib-0080] Wootton, R. J. (1994). Energy allocation in the threespine stickle‐back In The Evolutionary Biology of the Threespine Stickleback (Bell M. A. & Foster S. A., eds), pp. 116–143. Oxford: Science Publications.

[jfb13100-bib-0081] Wright, D. & Krause, J. (2006). Repeated measures of shoaling tendency in zebrafish (*Danio rerio*) and other small teleost fishes. Nature Protocols 1, 1828–1831. 17487165 10.1038/nprot.2006.287

[jfb13100-bib-0082] Bates, D. , Maechler, M. , Bolker, B. & Walker, S. (2014). lme4: Linear Mixed‐effects Models Using Eigen and S4. Vienna: R Foundation for Statistical Computing Available at http://www.r-project.org.

[jfb13100-bib-0083] Pinheiro, J. , Bates, D. , DebRoy, S. & Sarkar, D. (2007). Linear and Nonlinear Mixed Effects Models. Vienna: R Foundation for Statistical Computing Available at http://www.r-project.org.

