# Supplementary material for: Body length rather than routine metabolic rate and body condition correlates with activity and risk‐taking in juvenile zebrafish Danio rerio
Source: J Fish Biol. 2016 Sep 12;89(5):2251–67. doi: 10.1111/jfb.13100 (PMC6849769; doi:10.1111/jfb.13100)
Supplement: Supplementary file 1 — table SI. Results from the LMMs with distance moved, mean velocity, freezing and swimming in open water as dependent variables. Trial, L T (mm) and L T‐corrected residuals from the log‐transformed absolute RMR (mg O2 h−1) and log‐transformed Fulton's K (g mm−3 × 100 000) are included as fixed factors. Random intercepts are also included for each individual, which allowed variance decomposition. Intercepts (V among), residuals (V within) and adjusted repeatabilities are also shown with respect to each behavioural trait. Note that the fixed factor trial was consistently included for the analysis of the variance components: LMMs with one fixed factor per time (first three rows of the variance components) and LMM that includes all fixed factors (last row of the variance components) table SII. Results from the LMMs with distance moved, mean velocity, freezing and swimming in open water as dependent variables. Trial, body mass (g) and mass‐corrected residuals from the log‐transformed absolute RMR (mg O2 h−1) and log‐transformed Fulton's K (g mm−3 × 100 000) are included as fixed factors. Random intercepts are also included for each individual, which allowed variance decomposition. Intercepts (V among), residuals (V within) and adjusted repeatabilities are also shown with respect to each behavioural trait. Note that the fixed factor trial was consistently included for the analysis of the variance components: LMMs with one fixed factor per time (first three rows of the variance components) and LMM that includes all fixed factors (last row of the variance components) [file JFB-89-2251-s001.pdf]

Table SI.

Distance moved				
Fixed factors	Estimate	*F*	d.f.$_2$, d.f.$_1$	*P*
L_T	-2.94	4.62	4,5	0.04
RMR	11.59	2.54	4,5	0.13
K	-21.46	0.37	4,5	0.60
Trial	-0.78	0.03	4,5	0.86
Random factor		*F*	d.f.$_2$, d.f.$_1$	*P*
Individual		-	6,7	<0.001
Variance components		V_{within} ± S.E.	V_{among} ± S.E.	Repeatability
L_T		710.9 ± 2.3	720.6 ± 2.3	0.50
RMR		710.9 ± 2.3	751.3 ± 2.3	0.51
K		710.9 ± 2.3	785.2 ± 2.4	0.53
L_T, RMR, *K*		710.9 ± 2.3	675.6 ± 2.2	**0.49**

Mean velocity				
Fixed factors	Estimate	*F*	d.f.$_2$, d.f.$_1$	*P*
L_T	-0.79	0.77	4,5	0.38
RMR	-0.16	<0.01	4,5	0.98
K	15.94	0.52	4,5	0.48
Trial	-5.17	1.58	4,5	0.21
Random factor		*F*	d.f.$_2$, d.f.$_1$	*P*
Individual		-	6,7	0.05
Variance components		V_{within} ± S.E.	V_{among} ± S.E.	Repeatability
L_T		575.6 ± 2.1	160.7 ± 1.1	0.22
RMR		575.6 ± 2.1	165.8 ± 1.1	0.22
K		575.6 ± 2.1	162.4 ± 1.1	0.22
L_T, RMR, *K*		575.6 ± 2.1	157.4 ± 1.1	**0.21**

Freezing				
Fixed factors	Estimate	*F*	d.f.$_2$, d.f.$_1$	*P*
L_T	7.89	6.79	4,5	0.01
RMR	-27.25	2.82	4,5	0.11
K	73.64	0.90	4,5	0.41
Trial	-2.67	0.08	4,5	0.78
Random factor		*F*	d.f.$_2$, d.f.$_1$	*P*
Individual		-	6,7	<0.001
Variance components		V_{within} ± S.E.	V_{among} ± S.E.	Repeatability
L_T		3112 ± 5	3770 ± 5	0.55
RMR		3112 ± 5	4058 ± 5	0.57
K		3112 ± 5	4209 ± 6	0.57
L_T, RMR, *K*		3112 ± 5	3487 ± 5	**0.53**

Swimming in open water				
Fixed factors	Estimate	*F*	d.f.$_2$, d.f.$_1$	*P*
L_T	-0.07	2.58	4,5	0.12
RMR	0.06	0.04	4,5	0.84
K	-1.51	1.84	4,5	0.19
Trial	<0.01	<0.01	4,5	0.97
Random factor		*F*	d.f.$_2$, d.f.$_1$	*P*
Individual		-	6,7	0.03
Variance components		V_{within} ± S.E.	V_{among} ± S.E.	Repeatability
L_T		1.32 ± 0.10	0.44 ± 0.06	0.25
RMR		1.32 ± 0.10	0.48 ± 0.06	0.27
K		1.32 ± 0.10	0.45 ± 0.06	0.25
L_T, RMR, *K*		1.32 ± 0.10	0.41 ± 0.05	**0.24**

Table SII.

Distance moved				
Fixed factors	Estimate	*F*	d.f.$_2$, d.f.$_1$	*P*
Body mass	-213.34	5.39	4,5	0.03
RMR	12.12	2.42	4,5	0.14
K	34.72	0.85	4,5	0.45
Trial	-0.78	0.03	4,5	0.86
Random factor		*F*	d.f.$_2$, d.f.$_1$	*P*
Individual		-	6,7	<0.001
Variance components		V_{within} ± S.E.	V_{among} ± S.E.	Repeatability
Body mass		710.9 ± 2.3	713.5 ± 2.3	0.50
RMR		710.9 ± 2.3	753.6 ± 2.4	0.51
K		710.9 ± 2.3	780.3 ± 2.4	0.52
Body mass, RMR, *K*		710.9 ± 2.3	660.4 ± 2.2	**0.48**
Mean velocity				
Fixed factors	Estimate	*F*	d.f.$_2$, d.f.$_1$	*P*
Body mass	-48.78	0.65	4,5	0.42
RMR	0.17	<0.01	4,5	0.92
K	31.67	1.64	4,5	0.20
Trial	-5.17	1.58	4,5	0.21
Random factor		*F*	d.f.$_2$, d.f.$_1$	*P*
Individual		-	6,7	0.05
Variance components		V_{within} ± S.E.	V_{among} ± S.E.	Repeatability
Body mass		575.6 ± 2.1	161.5 ± 1.1	0.22
RMR		575.6 ± 2.1	165.7 ± 1.1	0.22
K		575.6 ± 2.1	155.1 ± 1.1	0.21
Body mass, RMR, *K*		575.6 ± 2.1	150.9 ± 1.1	**0.21**
Freezing				
Fixed factors	Estimate	*F*	d.f.$_2$, d.f.$_1$	*P*
Body mass	588.11	8.40	4,5	<0.01
RMR	-28.49	2.71	4,5	0.13
K	-74.62	0.81	4,5	0.48
Trial	-2.67	0.08	4,5	0.78
Random factor		*F*	d.f.$_2$, d.f.$_1$	*P*
Individual		-	6,7	<0.001
Variance components		V_{within} ± S.E.	V_{among} ± S.E.	Repeatability
Body mass		3112 ± 5	3683 ± 5	0.54
RMR		3112 ± 5	4070 ± 5	0.57
K		3112 ± 5	4224 ± 6	0.58
Body mass, RMR, *K*		3112 ± 5	3399 ± 5	**0.52**

Swimming in open water				
Fixed factors	Estimate	*F*	d.f.$_2$, d.f.$_1$	*P*
Body mass	-7.34	6.21	4,5	0.01
RMR	-0.07	0.05	4,5	0.83
K	-0.07	<0.01	4,5	0.94
Trial	<0.01	<0.01	4,5	0.97
Random factor		*F*	d.f.$_2$, d.f.$_1$	*P*
Individual		-	6,7	0.03
Variance components		V_{within} ± S.E.	V_{among} ± S.E.	Repeatability
Body mass		1.32 ± 0.10	0.38 ± 0.05	0.23
RMR		1.32 ± 0.10	0.48 ± 0.06	0.27
K		1.32 ± 0.10	0.48 ± 0.06	0.27
Body mass, RMR, *K*		1.32 ± 0.10	0.38 ± 0.05	**0.22**

6
